# Impact of in-hospital recurrent ischemia event: findings from GULF RACE-2

**DOI:** 10.1186/1753-6561-6-S4-O17

**Published:** 2012-07-09

**Authors:** A Al-Saleh, A Hersi, KF Alhabib, AA Alsheikh-Ali, K Sulaiman, H Alfaleh, S Alsaif, W Almahmeed, N Asaad, H Amin, A Al-Motarreb, J Al Suwaidi

**Affiliations:** 1 Department of Cardiac Sciences, College of Medicine, King Saud University, Riyadh, Saudi Arabia; 2 Department of Cardiac Sciences, Sheikh Khalifa Medical City, Abu Dhabi, United Arab Emirates; 3 Cardiology Department, Royal Hospital, Muscat, Oman; 4 Cardiology Department, Saud Al-Babtain Cardiac Center, Dammam, Saudi Arabia; 5 Department of Cardiology and Cardiovascular Surgery, Hamad Medical Corporation (HMC), Doha, Qatar; 6 Cardiology Department, Mohammed Bin Khalifa Cardiac Center, Manama, Bahrain; 7 Department of Medicine, Faculty of Medicine, Sana'a University, Sana'a, Yemen

## Introduction

Little in the literature is known about the long term outcome of patients with acute coronary syndrome (ACS) and in-hospital recurrent ischemic event. Accordingly; our objectives were to determine the baseline characteristics of patients, the predictors, and the long term outcome of patients with recurrent ischemia.

## Methods

The population compromised 7930 enrolled in the second Gulf Registry of Acute Coronary Events (Gulf RACE-2).

## Results

Out of the 7930 ACS patients, 172 (2.2%) had recurrent myocardial infarction (Re-MI) during their hospital stay. Patients with Re-MI were more likely to be older (mean age 59.12±13.5 vs. 56.8±12.4, P=0.016), had significantly higher rate of prior history of angina (48% vs. 38.2%, P=0.006), and hyperlipidemia (45.2% vs. 37.3%, P=0.027) than patients without Re-MI. On admission patients with Re-MI had significantly higher HR, lower systolic BP, Killip class 4 and high GRACE risk score than those without Re-MI (27.3% vs. 17.6%), (11% vs. 4.8%), (8.1% vs. 3.2%), and (31.8% vs. 21.5%, P<0.05 for all comparisons) respectively. Patients with Re-MI had a higher rate of STEMI on admission than patients without Re-MI (72.1% vs. 43.9%; P<0.001). Re-MI patients were less likely to receive Aspirin (94.8% vs. 98.5%, P=0.002), beta- blockers (95.3% vs. 74.7%, P<0.001), and Statin (87.2% vs. 94.9%, P<0.001) than patients without Re-MI. Coronary angiogram was less frequently performed on patients with Re-MI than patients without Re-MI (30.8% vs. 32.5%, P=0.036). In hospital adverse events including HF, cardiogenic shock, VT/VF were more frequent in the Re-MI group than patients without Re-MI (44.2% vs. 12.4%), (25.6% vs. 5.3%), (7.6% vs. 2.7%; P<0.001 for all comparisons) respectively. In ACS patients with Re-MI in-hospital, 30 days and 1 year were significantly higher that patients without Re-MI (23.8% vs. 4.1%), (28.1% vs. 7.7%), and (31.6% vs. 12.1%; P<0.001 for all comparisons), respectively.

## Conclusions

Recognizing patients at high risk of Re- MI is important as modifying the risk factors, and managing the patient aggressively may reduce the incidence of such events and the associated morbidity and mortality.

